# Visualizing Phonotactic Behavior of Female Frogs in Darkness

**DOI:** 10.1038/s41598-017-11150-y

**Published:** 2017-09-05

**Authors:** Ikkyu Aihara, Phillip J. Bishop, Michel E. B. Ohmer, Hiromitsu Awano, Takeshi Mizumoto, Hiroshi G. Okuno, Peter M. Narins, Jean-Marc Hero

**Affiliations:** 1 Graduate School of Systems and Information Engineering, University of Tsukuba, Tsukuba, Japan; 2 Department of Zoology, University of Otago, Dunedin, New Zealand; 3 Department of Biological Sciences, University of Pittsburgh, Pennsylvania, USA; 4 Graduate School of Informatics, Kyoto University, Kyoto, Japan; 5 Graduate Program for Embodiment Informatics, Waseda University, Tokyo, Japan; 6 Department of Integrative Biology & Physiology, University of California Los Angeles, California, USA; 7 Environmental Futures Research Institute, Griffith University, Gold Coast, Australia

## Abstract

Many animals use sounds produced by conspecifics for mate identification. Female insects and anuran amphibians, for instance, use acoustic cues to localize, orient toward and approach conspecific males prior to mating. Here we present a novel technique that utilizes multiple, distributed sound-indication devices and a miniature LED backpack to visualize and record the nocturnal phonotactic approach of females of the Australian orange-eyed tree frog (*Litoria chloris*) both in a laboratory arena and in the animal’s natural habitat. Continuous high-definition digital recording of the LED coordinates provides automatic tracking of the female’s position, and the illumination patterns of the sound-indication devices allow us to discriminate multiple sound sources including loudspeakers broadcasting calls as well as calls emitted by individual male frogs. This innovative methodology is widely applicable for the study of phonotaxis and spatial structures of acoustically communicating nocturnal animals.

## Introduction

Nocturnal animals use sounds for a variety of purposes. Bats emit ultrasounds to identify surrounding objects such as prey and obstacles^1^; barn owls localize moving prey with a high spatial resolution by analyzing interaural time and level differences of incoming sounds^2, 3^; crepuscular deer species vocalize for anti-predator purposes as well as for territory maintenance^4^. Thus, receiving and processing auditory information plays a crucial role for such nocturnal animals to survive in the wild.

Females of many species of anuran amphibians (frogs and toads) and insects exhibit positive phonotaxis toward vocalizing conspecific males prior to mating^5, 6^. In many species, males produce calls from a fixed location, and the female approaches the calling male by localizing his calls. Consequently, females are required to discriminate the qualities of the males based on features of their calls^5, 6^. Such phonotaxis has been investigated in playback experiments using loudspeakers, demonstrating that female anurans and insects show acoustic preferences depending on the call features of conspecific males, such as call frequency, call duration, call complexity and leader-follower relationship^7–11^. In contrast, phonotactic behaviour of female anurans and insects in their natural habitats is poorly known due to (1) the presence of multiple individual calling males whose positions and call timing are unknown, and (2) females are cryptic and move around silently through a large and vegetatively complex area, making it difficult to reliably track their positions.

**Figure 1:**
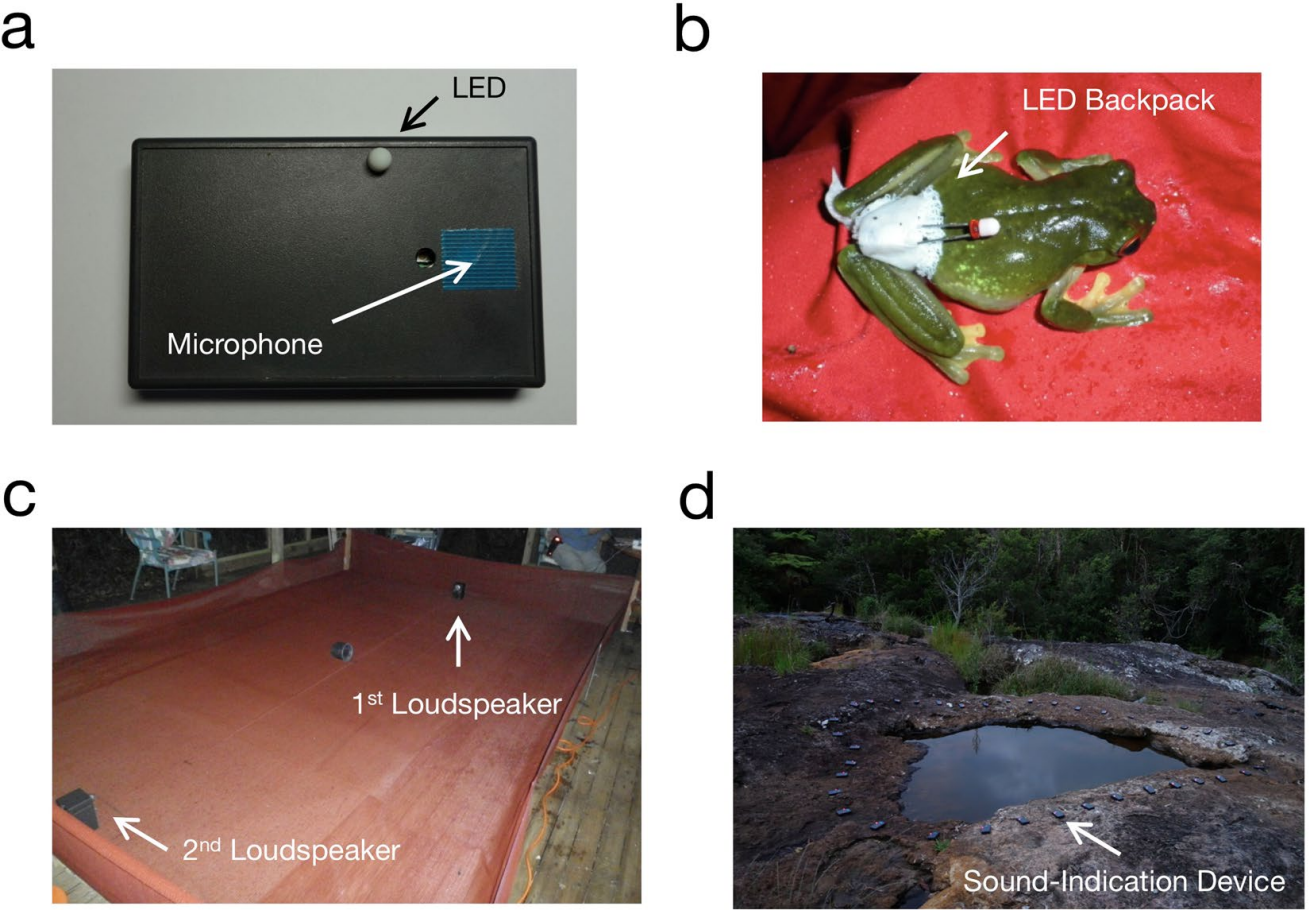
Playback experiments with, and field recordings of the phonotactic behavior of female frogs (Australian orange-eyed tree frogs (*Litoria chloris*)). (**a**) Sound-indication device *Firefly*. The *Firefly* unit is illuminated when detecting nearby sounds. (**b**) Female frog with a miniature backpack consisting of an LED and a button battery. (**c**) Setup of the arena playback experiments. A loudspeaker was placed at each end of the arena. (**d**) Field site at Springbrook National Park, Queensland, Australia. Male frogs were chorusing along the edge of this pool.

To solve the problem of localization and separation of multiple sound sources, a sophisticated audio-recording system such as a microphone array system is useful. For example, the positions of echolocating bats and dolphins have been estimated from the time difference of arrival between several pairs of microphones^12, 13^. Spatio-temporal structures of frog choruses have also been estimated using such a microphone array system^14–16^. While a microphone-array system provides a precise estimate of caller positions and call timing, it is expensive and also requires significant time and effort to deploy. For example, the microphones require long cables, and distances between each pair of the microphones must be precisely measured. In contrast, Mizumoto *et al*. (2011) proposed an inexpensive and tractable system for sound source localization based on a sound-indication device called *Firefly* (Fig. 1a)^17^. The *Firefly* unit consists of a miniature microphone and a light emitting diode (LED) that is illuminated when detecting nearby sounds. Dozens of these devices were deployed at a natural breeding site of the Japanese tree frog (*Hyla japonica*). The illumination patterns of the devices were then recorded with an off-the-shelf video camera. Analysis of the video demonstrated that the caller positions and call timing of several male frogs were precisely estimated^17, 18^.

In this study, we propose a novel technique for visualizing phonotaxis of nocturnal animals by combining the *Firefly* system with a tracking technique using a miniature LED backpack (Fig. 1b). With this system, we were able to visualize the movement of females of the Australian orange-eyed tree frog (*Litoria chloris*) towards conspecific males. To our knowledge, this is a novel system for simultaneously examining the trajectory of female frogs as well as the calling behavior of conspecific males in their natural habitat. The technique is relatively inexpensive and easily deployable for the study of phonotaxis in nocturnal animals such as anurans, making it a substantial methodological advancement for the field.

## Results

### Arena Playback Experiments

To visualize the phonotaxis of the female frogs (*L*. *chloris*) in a laboratory arena, we conducted playback experiments using a miniature LED backpack, sound-indication devices, and loudspeakers. One loudspeaker was placed at each end of the arena (Fig. 1c). Higher-frequency and lower-frequency calls of male *L*. *chloris* were broadcast through the 1^st^ and 2^nd^ loudspeakers, respectively. A sound-indication device (*Firefly*) was placed about 6 cm in front of each loudspeaker. A miniature backpack consisting of an LED and a button battery was mounted on the female frogs under test. One female frog was placed in a small mesh cage for 3 min at the center of the arena, and then released from that position. The lights of the *Firefly* devices and miniature backpack were recorded by an off-the-shelf video camera that was fixed on a tripod.

**Figure 2:**
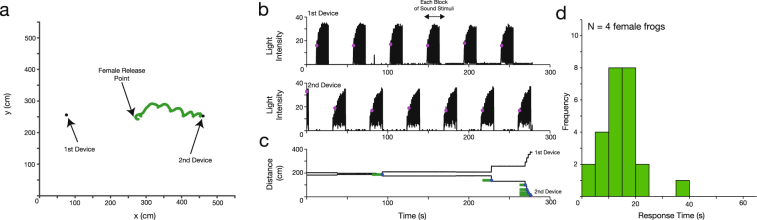
Playback experiments for visualizing the phonotactic behavior of female frogs. (**a**) Trajectory of a female frog approaching one of the loudspeakers. The green line represents the position of a female frog. Black dots represent the positions of the sound-indication devices that were placed about 6 cm in front of each loudspeaker. (**b**) Illumination pattern of the sound-indication devices. Pink dots represent onsets of each block of sound stimuli that consist of male *L*. *chloris* calls. (**c**) Distance from a female frog to each device. Blue dots represent timing when the female hopped towards one of the loudspeakers. Comparison between the illumination pattern and distance to the devices allows us to estimate response time (green bars) that this female frog required to make a decision to approach one of the loudspeakers. (**d**) Histogram of the response times obtained from playback experiments (N = 4 female frogs).

Analysis of the video revealed the trajectory of female frogs and the timing of the sound stimuli. Figure 2 shows a representative result of the playback experiments, in which a female frog reached the 2^nd^ loudspeaker 4 min 36 s after her release (Fig. 2a and c). The illumination pattern of the *Firefly* devices demonstrated that each block of the sound stimuli consisting of the calls of males of *L*. *chloris* was played alternately between the two loudspeakers (Fig. 2b). The time series of the distance between a female frog and each sound-indication device was then calculated, allowing us to estimate the precise time when a female hopped toward one of the loudspeakers (Fig. 2c). The time interval between the onset of the stimulus and a hop was considered the time required by the female to make a decision to approach the focal loudspeaker (see green bars in Fig. 2c).

Figure 2d shows the histogram of response times required for decision making for females of *L*. *chloris* (N = 4 frogs). The times that females took until reaching one of the loudspeakers ranged from 2 min 33 s to 7 min 58 s; the backpack remained affixed to the females in all trials. The response times (N = 25) were estimated at 13.74 ± 6.99 s (mean ± SD). In our playback experiments, two female frogs reached the loudspeaker that was emitting higher-frequency calls while the other two frogs reached the loudspeaker that was emitting lower-frequency calls.

### Field Recordings

We applied the imaging and tracking methodology used for our playback experiments to field recordings, to visualize the phonotaxis of females of *L*. *chloris* in their natural habitat. Males of *L*. *chloris* were chorusing along the edge of a rock pool in our study site (Fig. 1d). To analyze their chorus structures, 28 sound-indication devices were deployed along the edge of the pool with an average spacing between adjacent units of 35 cm. A miniature LED backpack was mounted on each female frog, just as in our arena playback experiments. Each female frog with the miniature LED backpack was kept in a small mesh cage for 5 min besides the pool, and then released at the same position. The lights of the backpack and sound-indication devices were recorded by the same video camera used for the arena-playback experiments.

**Figure 3:**
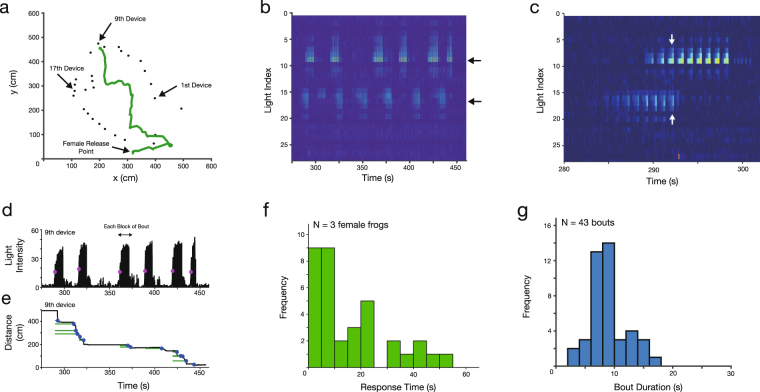
Field recordings for visualizing the phonotactic behavior of female frogs. (**a**) Trajectory of a female frog. (**b**) Illumination pattern of sound-indication devices. Two male frogs were chorusing in the vicinity of the 9^th^ and 17^th^ devices, respectively. (**c**) Discrimination of overlapping calls. As depicted by the arrows, our imaging methodology allows us to discriminate overlapping calls of two male frogs. (**d**) Illumination pattern of the 9^th^ device. (**e**) Distance from a female frog to the 9^th^ device. This female frog reached the 9^th^ device 7 min 23 s after her release. Blue dots represent timing when the female hopped towards a male frog at the 9^th^ device, and green bars represent the response time of this female frog. (**f**) Histogram of the response times obtained from field recordings (N = 3 female frogs). (**g**) Histograms of the bout durations obtained from field recordings (N = 43 bouts).

Video analysis revealed the trajectory of female frogs as well as the call properties of male frogs around the rock pool where multiple male frogs (at least two frogs) were chorusing. Figure 3 shows a representative field recording; this female reached the 9^th^ *Firefly* device 7 min 23 s after her release (Fig. 3a and e). The illumination pattern of the *Fireflies* demonstrated that two male frogs were chorusing in the vicinity of the 9^th^ and 17^th^ devices, respectively (Fig. 3b and c). Consequently, it was shown that the female frog chose the male frog nearest to the 9^th^ device for mating. As reported in a previous study^17^, the *Firefly* devices allow us to discriminate overlapping calls of two male frogs (Fig. 3c). By analyzing the illumination pattern of the 9^th^ device (Fig. 3d) as well as the distance from the female to the 9^th^ device (Fig. 3e), we estimated the time interval that the female frog required to respond to the calling bout of the focal male frog (see green bars in Fig. 3e). This interval is defined as the time between the stimulus onset just prior to and closest to each hop of a female frog, and the time of her hop.

Figure 3f shows the histogram of response times of females of *L*. *chloris* in the animal’s natural habitat (N = 3 frogs). The time that females took until reaching a calling male frog ranged from 7 min 23 s to 13 min 26 s; the backpack remained affixed to the females in all trials. The response times (N = 35) were estimated at 16.17 ± 14.62 s (mean ± SD).

### Relationship between Call Properties and Response Time

To examine the effect of call properties of the male frogs on response times of the females, we calculated the duration of sound stimuli as well as that of actual bouts. From the arena playback experiments, the duration of sound stimuli (see the arrow in Fig. 2b) was estimated at 13.2 ± 0.5 s (mean ± SD, N = 25 stimulus blocks). These values are close to those of response times of female frogs obtained from the same experiments (13.7 ± 7.0 s, N = 25 response times), indicating that the females make efforts to hear whole bouts and make their decisions of approach during the arena playback experiments.

We then calculated the bout duration of actual male frogs (see the arrow in Fig. 3d) by using the illumination pattern of the particular sound-indication device that female frogs finally reached. The bout duration was estimated at 8.9 ± 3.0 s (mean ± SD, N = 43 bouts, Fig. 3g). On the other hand, the response times of the female frogs were 16.17 ± 14.62 s (mean ± SD, N = 35 response times). Although the mean value of the response time is larger than that of the bout durations, they are not significantly different (P = 0.38, Brunner-Munzel test).

### Discrimination of Call Frequency

**Figure 4:**
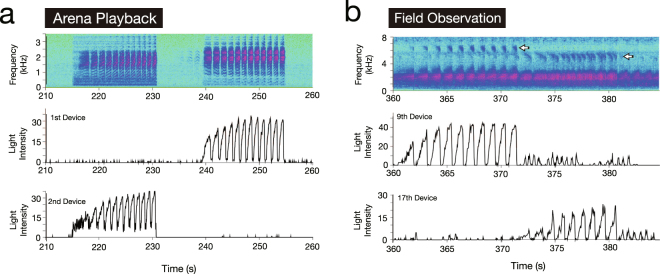
Discrimination of call frequency from (**a**) arena playback experiments and (**b**) field recordings. Top panels represent the spectrograms of audio data recorded by a video camera. Middle and bottom panels represent the illumination patterns of the sound-indication devices closest to the respective sound sources (i.e., loudspeakers or calling male frogs). Comparison between the spectrograms and illumination patterns allows us to discriminate call frequency of the respective sound sources. Figure 4a and b were obtained from the same dataset shown in Figs 2a–c and 3a–e, respectively.

To discriminate the frequency of the sound sources, we compared the illumination pattern of sound-indication devices with the spectrogram of the audio data recorded by the video camera according to the method proposed by Aihara *et al*.^19^. Figure 4a depicts the illumination patterns and spectrogram recorded during a playback experiment (corresponding to the experiment shown in Fig. 2a–c), demonstrating that higher-frequency calls were broadcast through the 1^st^ loudspeaker, while lower-frequency calls were broadcast through the 2^nd^ loudspeaker (Fig. 4a). This result is consistent with the frequency of the calls used for our playback experiments (see Methods).

We then examined the frequencies of frog calls using the audio data from a field experiment corresponding to the data shown in Fig. 3a–e. The spectrograms tended to be noisy around 2 kHz, which included the dominant frequency in the calls of males of *L*. *chloris*. As a result, we focused on frequencies in the range of 5 kHz, which included the second harmonics of the calls (see arrows in Fig. 4b). Comparison of the spectrograms with the illumination patterns of the 9^th^ and 17^th^ devices (devices closest to the respective calling frogs) demonstrated that the male frog at the 9^th^ device vocalized with relatively high-frequency calls while the frog at the 17^th^ device vocalized with relatively low-frequency calls (Fig. 4b). Since the female frog approached and reached the male frog nearest to the 9^th^ device (Fig. 3a), this female preferred higher-frequency calls in this trial.

## Discussion

This study demonstrates that phonotaxis of female frogs of *L*. *chloris* can be visualized using a miniature LED backpack and sound-indication devices both in a laboratory arena and in the animals’ natural habitat. The light on the backpack mounted on a female allows automatic tracking of her position (Figs 2a and 3a), and the illumination patterns of the sound-indication devices allow us to discriminate the timing of sound sources such as loudspeakers and calling males (Figs 2b and 3d). In addition, the comparison of the illumination patterns with the audio spectrogram allows the discrimination of the call frequency of two sound sources (Fig. 4), which is applicable to the analysis of other call features such as call complexity^19^ even in a dense chorus if there are non-overlapping calls. Thus, our work demonstrates a proof of concept; additional field recordings are required to identify the call parameters (e.g., call frequency and the number of calls included in each bout) associated with the acoustic preference of females of *L*. *chloris*.

Compared to related techniques, the proposed methodology will improve our understanding of female decision making in various ways: (1) the miniature backpack allows us to automatically and continuously track the phonotactic behavior of female frogs at a high spatio-temporal resolution (1440 × 1080 pixels and 29.97 fps in our case); (2) the spatio-temporal structures of frog choruses can be detected as illumination patterns of the *Firefly* devices even when overlapping calls exist; (3) the combination of these two techniques allows us to follow female movements and selection strategies under natural light conditions of a field site, while decreasing disturbance by the observer.

The position of a female frog is indicated by the light of an LED. Therefore, her position can be continuously detected and tracked by a video camera, even in their natural habitat where the distance between the camera and a moving female is continuously varying. Moreover, the *Firefly* devices allow the discrimination of overlapping calls via illumination patterns of different devices that are spatially separated (Fig. 3c)^17^. In contrast, it is well known that overlapping calls seriously deteriorate sound-source localization in a microphone array system. Our methodology would be applicable to the study of female movement and selection strategies of various nocturnal animals forming choruses (e.g., anuran amphibians and insects) because call overlaps occasionally happen in their choruses.

Several technical problems still remain to be addressed to perfect our methodology. For example, discrimination of multiple sound sources was not always possible. In the complex spatial structure of the rock-pool habitat, the light intensity of the *Firefly* LED changed nonlinearly with distance from a nearby sound source, thus precluding an accurate localization of male frogs in some choruses. Improvements in the response range of the *Fireflies* as well as in the microphone placement within a device, are currently in progress. In particular, if the *Fireflies* are miniaturized to the size of several cm and tuned to respond only to nearby sounds within several cm, we would be able to discriminate male frogs chorusing in a dense distribution by deploying improved devices at distances between units of several cm. Moreover, the use of multiple video cameras would allow the localization of frogs in a 3-dimensional space. The software developed for this study will be released in open-source format in the near future.

## Methods

### Study Site

Playback experiments and field recordings were conducted at Springbrook National Park (SNP), Queensland, Australia in 2012 and 2013. Playback experiments were performed within an arena (Length: 4.4 m, Width: 3.0 m, Height: 0.3–0.5 m) between 21:50 h and 01:40 h (next day) in February, 2012. The floor and walls of the arena were made of terracotta-coloured 70% nylon shade cloth. The temperature of the arena was 18 °C. Field recordings were performed at a rock pool beside a stream in the SNP (28°11′43.30″ S, 153°16′3.69″ E) between 21:00 h and 22:40 h in January, 2013. The temperature of the field site was 23 °C.

### Materials

Australian orange-eyed tree frogs, *Litoria chloris*, were used in both the arena playback experiments and field recordings. *L*. *chloris* is a stream-breeding hylid species found in the rainforests along the coastlines of Queensland and New South Wales in eastern Australia. Male frogs produce advertisement calls, and female frogs use these calls to discriminate between males and approach a potential mate. Male frogs periodically produce the calls at 1-s intervals and each calling bout consists of about ten successive calls (Fig. 4b)^20^.

Female frogs used for this study were obtained from amplectant pairs, and were representative of the size of this population (SUL: 67.3–73.9 mm (Playback experiments, N = 4 female frogs), 61.5–76.2 mm (Field recordings, N = 3 female frogs)). Following the playback experiments and field recordings, all the female frogs resumed amplexus with their specific mates, and pairs were released at the same locations where they were captured.

### Playback experiments

To investigate the phonotaxis of females of *L*. *chloris* and test the effectiveness of our methodology for automatic tracking of female frogs, we conducted playback experiments using two loudspeakers (Tivoli PAL) placed at opposite ends of the arena, 4 m apart. The LED of a *Firefly* unit^17^ was placed about 6 cm in front of each loudspeaker. Higher-frequency and lower-frequency calls of males of *L*. *chloris* (Dominant frequencies: 1898 Hz and 1690 Hz, corresponding to small and large males, respectively^21^) were digitally broadcast (Apple 3 G iPod Nano) through the 1^st^ and 2^nd^ loudspeakers, respectively (Figs. 1c and 2a). Sound pressure levels of the two sound stimuli were set at 90 dB SPL at 50 cm from their respective loudspeakers by using a sound level meter (IEC 651 Type 2, Extech Instruments; RMS fast, C-weighting). Each block of the sound stimuli consisted of 12 calls (termed a “bout”), which were repeatedly broadcast with an intervening silent period of 30 s.

The miniature backpack, which consists of an LED and a button battery, was attached to female frogs by a temporary belt constructed using medical tape (3 M Micropore Medical Surgical Paper Tape Brown Sensitive (1.27 cm wide)) according to an approved method^22^. Prior to the attachment, we carefully folded the adhesive surface of the tape inward to prevent adhesive contact with the skin of the test animals. The total weight of the backpack and belt was 1.8 g. Individual female frogs were placed in a mesh cage at the center of the arena for 3 min, during which the sound stimuli were broadcast to habituate the females to these stimuli^23^. The female frog was then released from the cage at the same position. The lights of the miniature backpack and sound-indication devices were recorded by an off-the-shelf video camera (Sony, HDR-XR550V) at a sampling rate of 29.97 fps (frames per second), a resolution of 1440 × 1080 pixels for video recordings, and 48 kHz for audio recordings. The video camera was placed in a fixed position on a tripod (Sony, VCT-80AV) during the experiments. In addition, we carefully monitored the behavior of female frogs with night-vision goggles and did not observe any unusual behaviors or signs of stress.

Each experiment was completed when one of the following conditions had been satisfied: (1) a female frog reached within 10 cm of one of the loudspeakers within a 10-min trial, (2) a female frog hopped out of the arena, or (3) 10 min had passed without satisfying either of the first two conditions. For the second condition, we repeated the same experiment one more time using the same frog. The miniature backpack was only attached less than 30 minutes for each frog.

We cut the medical cape with scissors immediately after each experiment and removed the backpack from female frogs. We confirmed that no frog was injured by this process. In addition, we conducted the same experiments without the miniature backpack and carefully monitored the behavior of female frogs by using night vision goggles^24^. By comparing the behavior of female frogs with and without the backpack, we are confident that the female frogs suffered no adverse effects from this procedure.

### Field recordings

To examine the phonotaxis of females of *L*. *chloris* in their natural habitat, the methodology of the arena playback experiments was applied in the field. To simultaneously estimate caller positions and call timing, 28 *Firefly* devices were deployed along the edge of the rock pool as shown in Fig. 1d. Field-tracking of female frogs was conducted using similar methodology to that of the arena playback experiments except a lighter miniature LED backpack and a non-adhesive medical tape were used. Individual female frogs were kept in a mesh cage for 5 min besides the pool, and were then released at the same position. The initial positions of female frogs were set near the edge of the pool, and as equally distant from the calling males around the pool as possible. The lights of the backpack and sound-indication devices were recorded by a video camera (Sony, HDR-XR550V) that was fixed on a tripod (Sony, VCT-80AV). During our recordings, multiple male frogs (at least two male frogs) were calling along the edge of the rock pool. Given the higher background noise levels in the field compared to the playback arena, females in the field experiments were allowed to habituate to the background noise for 5 min, compared to 3 min for the females in the arena playback experiments.

Each experiment was completed when a female frog amplexed with a calling male frog. After amplexus was achieved, we released the male frog chosen by the female frog at the same position where he was calling.

### Analysis of video data

Video analysis of both arena playback and field experiments consisted of estimating the position and timing of each sound source (i.e., loudspeaker and calling male frog, respectively) as well as the positions of female frogs. The illumination patterns of the sound-indication devices were calculated according to the method of Mizumoto *et al*.^17^; the video data was divided into still frames at the rate of 29.97 frames per second; a number of frames (50 frames for arena experiments and 150 frames for field recordings) were summed; the position of an LED attached to each device was estimated as blocks of bright pixels in the summed-up frame; the time series of the brightness of each device was extracted using its LED position. Each female frog was tracked by calculating the brightest pixel in each frame, after discounting the lights of the sound-indication devices.

The positions of the sound-indication devices and females were converted from coordinates in still frames (pixel no.) to those in actual space (cm) based on the method of homography using the video data of a single camera (see Supplementary Information)^25^. We first chose four points whose coordinates are known both in the frames and actual space, and then calculated a conversion matrix from pixels to cm by using both coordinates of the four reference points. The positions of the sound-indication devices and females in actual space were obtained by multiplying the conversion matrix by the vector of their pixel coordinates. Note that this analysis assumes a 2-dimensional distribution of frogs (see Supplementary Information); the assumption is not perfect but still valid in our case because our arena was flat (see Fig. 1c) and the rock pool and its surroundings are reasonably flat (see Fig. 1d).

To quantify the features of sound sources, we analyzed the illumination patterns of the *Firefly* devices. According to the method proposed in a previous study^18^, we first detected call timings of the *i* ^th^ sound source as ${T}_{i}^{n}$ where *n* represents the index of calls. We then determined call bouts depending on an inter-call interval ${\rm{\Delta }}{T}_{i}^{n}={T}_{i}^{n+1}-{T}_{i}^{n}$. Namely, the *n* ^th^ and *n* + 1^th^ calls are determined to be included in the same bout if ${\rm{\Delta }}{T}_{i}^{n}$ is less than a threshold value of 5 s that is sufficiently larger than a typical value of inter-call intervals of male *L*. *chloris*. Subsequently, we estimated onsets of calling bouts and the durations of calling bouts.

The response time of female frogs was estimated from the illumination patterns and female positions in actual space. According to the analysis described in the above paragraph, onsets of calling bouts were detected as the timing of the first call of each calling bout (Figs 2b and 3d). We then calculated the distance from a female frog to the *i* ^th^ sound source as *r*_*i*_(*t*). The change of *r*_*i*_(*t*) between adjacent frames was then calculated as Δ*r*_*i*_(*t*) = *r*_*i*_(*t*) − *r*_*i*_(*t* + 1). When Δ*r*_*i*_(*t*) exceeded a positive threshold value of 3.5 cm that corresponds to almost a half the snout-vent length of females of *L*. *chloris*, the female frog was determined to have hopped towards the *i* ^th^ sound source. Consequently, the response time of female frogs was estimated as the time interval from the onset of the previous calling bout to the hop.

### Analysis of the audio data

Audio data was extracted from each video. The spectrogram of the audio data was calculated by using the *spectrogram* function of MATLAB (version 8.3.0.532 (R2014a)).

### Ethical approval

All the methods were carried out in accordance with the guidelines of the QPWS Scientific Research and the Griffith University Animal Ethic Committee. All the experimental protocols were approved by QPWS Scientific Research Permit #WITK13676013, and the Griffith University Animal Ethic Committee Reference # ENV/25/11 and ENV/20/13/AEC.

## Electronic supplementary material

Supplementary Manuscript

## References

[1] Griffin, D. R. *Listening in the Dark: The Acoustic Orientation of Bats and Men*. (Yale University Press, 1958).

[2] Knudsen, E. I. & Konishi, M. Mechanisms of sound localization in the barn owl (*Tyto alba*). *J. Comp. Physiol. A* **133**, 13–21 (1979).

[3] Knudsen EI (1981). The hearing of the barn owl. Scientific American.

[4] Reby D, Cargnelutti D, Hewison AJM (1999). Contexts and possible functions of barking in roe deer. Anim. Behav..

[5] Gerhardt, H. C. & Huber, F. *Acoustic Communication in Insects and Anurans*. (University of Chicago Press, 2002).

[6] Wells, K. D. *The Ecology and Behavior of Amphibians*. (The University of Chicago Press, Chicago, 2007).

[7] Snedden WA, Greenfield MD (1998). Females prefer leading males: relative call timing and sexual selection in katydid choruses. Anim. Behav..

[8] Gerhardt, H. C., Tanner, S. D., Corrigan, C. M. & Walton, H. C. Female preference function based on call duration in the gray tree frogs (*Hyla versicolor*). *Behav. Ecol.* **11**(6), 663–669 (2000).

[9] Jennions, M. D., Backwell, P. R. Y. & Passmore, N. I. Repeatability of mate choice: the effect of size in the African painted reed frog, *Hyperolius marmoratus*. *Anim. Behav.* **49**, 181–186 (1995).

[10] Rand AS, Ryan MJ (1981). The adaptive significance of a complex vocal repertoire in a neotropical Frog. Zeitschrift für Tierpsychol.

[11] Lopez, P. T. & Narins, P. M. Mate choice in the neotropical frog, *Eleutherodactylus coqui*. *Anim. Behav.* **41**, 757–772 (1991).

[12] Fujioka E (2014). Rapid shifts of sonar attention by Pipistrellus abramus during natural hunting for multiple prey. J. Acoust. Soc. Am..

[13] Au, W. W. L. & Herzing, D. L. Echolocation signals of wild Atlantic spotted dolphin (*Stenella frontalis*). *J. Acoust. Soc. Am.* **113**, 598–604 (2003). 10.1121/1.1518980 12558295

[14] Grafe, T. U. Costs and benefits of mate choice in the lek-breeding reed frog, *Hyperolius marmoratus*. *Anim. Behav.* **53**, 1103–1117 (1997).

[15] Simmons, A. M., Simmons, J. A. & Bates, M. E. Analyzing acoustic interactions in natural bullfrog (*Rana catesbeiana*) choruses. *J. Comp. Psychol.* **122**, 274–282 (2008). 10.1037/0735-7036.122.3.274 PMC2556862 18729655

[16] Jones DL, Jones RL, Ratnam R (2014). Calling dynamics and call synchronization in a local group of unison bout callers. J. Comp. Physiol. A.

[17] Mizumoto T (2011). Sound imaging of nocturnal animal calls in their natural habitat. J. Comp. Physiol. A.

[18] Aihara I (2014). Spatio-temporal dynamics in collective frog choruses examined by mathematical modeling and field observations. Sci. Rep..

[19] Aihara I, Silva Pde, Bernal XE (2016). Acoustic preference of frog-biting midges (Corethrella spp) attacking túngara frogs in their natural habitat. Ethology.

[20] MacLean, M. J., Bishop, P. J., Hero, J.-M. & Nakagawa, S. Assessing the information content of calls of *Litoria chloris*: quality signalling versus individual recognition. *Australian Journal of Zoology* **60**, 120–126 (2012).

[21] Morrison, C., Hero, J.-M. & Smith, W. Mate selection by female *Litoria chloris* and *L. xanthomera*: size doesn’t always matter. *Austral Ecology* **26**(3), 223–245 (2001).

[22] Hodgkison, S. & Hero, J.-M. Daily behaviour and microhabitat use of the Waterfall frog, *Litoria nannotis*, in Tully Gorge, eastern Australia. *Journal of Herpetology* **35**, 116–120 (2001).

[23] Narins, P. M. & Capranica, R. R. Communicative significance of the two-note call of the treefrog *Eleutherodactylus coqui*. *J. Comp. Physiol. A* **127**, 1–9 (1978).

[24] Bishop, P. J., Narins, P. M., Aihara, I., Ohmer, M. E. B. & Hero, J.-M. Successful sexy small males ignore female preference in the red-eyed tree frog (*Litoria chloris*), Abstracts of a Satellite Symposium of the 15^th^ Congress of the International Society for Behavioral Ecology, 4 (2014).

[25] Solem J. E. *Programming Computer Vision with Python*. (O’Reilly Media, 2012).

