# Supplementary material for: Visualizing Phonotactic Behavior of Female Frogs in Darkness
Source: Sci Rep. 2017 Sep 5;7:10539. doi: 10.1038/s41598-017-11150-y (PMC5585358; doi:10.1038/s41598-017-11150-y)
Supplement: Supplementary file 1 — Supplementary Manuscript [file 41598_2017_11150_MOESM1_ESM.pdf]

Supplementary Information:
Visualizing Phonotactic Behavior of Female Frogs in Darkness

Ikkyu Aihara[1]*, Phillip J. Bishop[2], Michel E. B. Ohmer[3], Hiromitsu Awano[4], Takeshi Mizumoto[4], Hiroshi G. Okuno[5], Peter M. Narins[6], Jean-Marc Hero[7]

[1] Graduate School of Systems and Information Engineering, University of Tsukuba, Tsukuba, Japan
[2] Department of Zoology, University of Otago, Dunedin, New Zealand
[3] Department of Biological Sciences, University of Pittsburgh, Pennsylvania, USA
[4] Graduate School of Informatics, Kyoto University, Kyoto, Japan
[5] Graduate Program for Embodiment Informatics, Waseda University, Tokyo, Japan
[6] Department of Integrative Biology & Physiology, University of California Los Angeles, California, USA
[7] Environmental Futures Research Institute, Griffith University, Gold Coast, Australia

*Corresponding Author:
aihara@cs.tsukuba.ac.jp

Coordinate Transformation using Homography

Based on the technique of homography assuming that target objects are positioned in a 2-dimensional space[1], we convert the coordinates of pixels to those of an actual space. Consider the following problem converting one point $\vec{r}$ to another point $\vec{R}$ in a two-dimensional space by using a conversion matrix H and a scale factor w:

$$w\vec{R} = H\,\vec{r}, \tag{1}$$

with

$$\vec{r} = \begin{pmatrix} x \\ y \\ 1 \end{pmatrix}, \tag{2}$$

$$\vec{R} = \begin{pmatrix} X \\ Y \\ 1 \end{pmatrix}, \tag{3}$$

$$H = \begin{pmatrix} h_{11} & h_{12} & h_{13} \\ h_{21} & h_{22} & h_{23} \\ h_{31} & h_{32} & 1 \end{pmatrix}, \tag{4}$$

When analyzing our data, we assume that $\vec{r}$ and $\vec{R}$ correspond to the points of pixels and those of an actual space, respectively. The important concept is that a conversion matrix H includes eight parameters of h_{11}, h_{12}, h_{13}, h_{21}, h_{22}, h_{23}, h_{31}, and h_{32}. Because a pair of the coordinates $\vec{r}$ and $\vec{R}$ gives two equations with respect to these parameters, we need four sets of $\vec{r}$ and $\vec{R}$ in order to determine the parameter values.

To estimate a conversion matrix H, we choose four reference points whose coordinates are known both in pixels and actual space (see Figure S1). We describe the pixel coordinates as $\vec{r}_1$, $\vec{r}_2$, $\vec{r}_3$ and $\vec{r}_4$, and the actual coordinates as $\vec{R}_1$, $\vec{R}_2$, $\vec{R}_3$ and $\vec{R}_4$. Substituting $\vec{r}_n$ and $\vec{R}_n$ $(n = 1, 2, 3, 4)$ into Eq.(1) gives eight equations related to the unknown parameters, which allows us to calculate a conversion matrix H. Consequently, we obtain the actual coordinates of sound-indication devices and LED backpack by multiplying H to their pixel coordinates $\vec{r}$ according to Eq.(1).

During arena playback experiments and field observations, we recorded dim

lights of sound-indication devices and LED backpack in darkness, which makes it difficult to find the same reference points for all the experiments. Therefore, we first carefully looked at all the video data, and chose the brightest picture where we can obtain more than four reference points. The actual coordinates of those points were calculated according to the technique of homography. When analyzing the remaining datasets, we use four of the reference points as $\vec{R}_1$, $\vec{R}_2$, $\vec{R}_3$ and $\vec{R}_4$, and re-calculated a conversion matrix H. This re-calculation was required because the angle of a video camera slightly changed in respective experiments (when we pushed control buttons of the camera, the angle of the camera slightly changed).

Figure S1 shows an example of the conversion on our arena playback experiments, demonstrating that the pixel coordinates of sound-indication devices and LED backpack are converted to the coordinates in the actual space.

Reference

1. Solem J.E. *Programming Computer Vision with Python*. (O'Reilly Media, 2012).

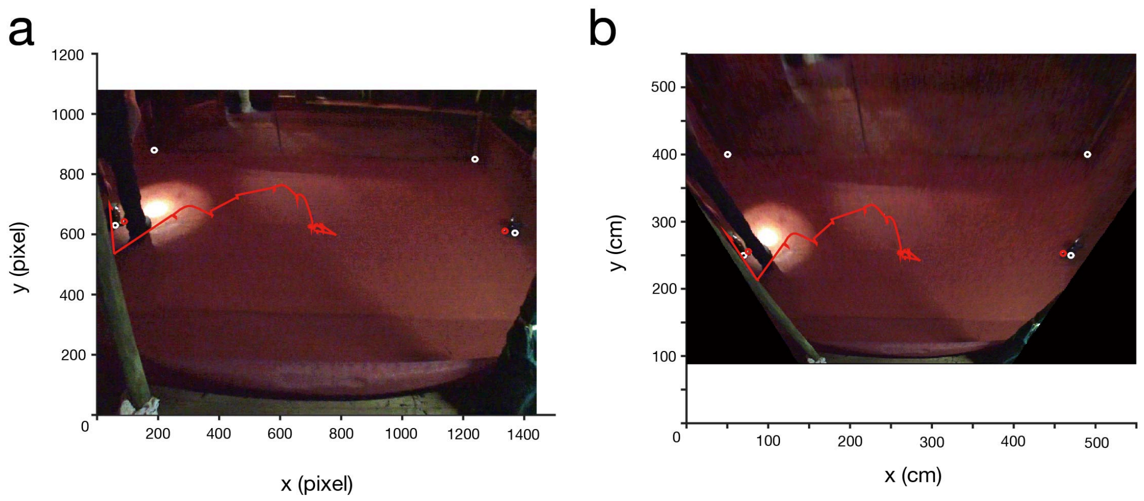

Figure S1| Conversion of pixel coordinates to actual coordinates. (a) Coordinates in pixels. (b) Coordinates in an actual space. White circles represent reference points that are used for the calculation of a conversion matrix H. Red circles and lines are the coordinates of sound-indication devices and LED backpack, respectively.
